# Cross-neutralising antibodies to SARS-CoV-2 in sera from straw-coloured fruit bats (*Eidolon helvum*) in Nigeria

**DOI:** 10.1186/s12917-025-04938-y

**Published:** 2025-08-29

**Authors:** Hooman Goharriz, Guanghui Wu, Veronica O. Ameh, Amanda H. Seekings, Joan Amaya-Cuesta, Lorraine M. McElhinney, Claude T. Sabeta

**Affiliations:** 1 Viral Zoonoses Group, Animal and Plant Health Agency (APHA), Woodham Lane Weybridge, New Haw, Surrey, Addlestone, KT15 3NB UK; 2 Department of Veterinary Public Health and Preventive Medicine, College of Veterinary Medicine, Joseph Sarwuan Tarka University Makurdi, P.M.B, Makurdi, 2373 Benue State Nigeria; 3 Institute of Infection, Veterinary and Ecological Sciences, University of Liverpool, IC2 Building, Liverpool Science Park, 146 Brownlow Hill, Liverpool, L3 5RF UK; 4 Department of Veterinary Tropical Diseases, Faculty of Veterinary Science, University of Pretoria, P Bag X04, Onderstepoort, 0110 South Africa

**Keywords:** *Eidolon helvum*, Bat coronavirus, Lineage D beta coronavirus, SARS-CoV-2, Virus neutralisation test

## Abstract

**Background:**

Straw-coloured fruit bats (*Eidolon helvum; *) are widely distributed in Africa and are known reservoirs for viruses with zoonotic potential. These bats are widely hunted in West and Central Africa for human consumption as food source and medicine. This practice increases the potential for spillover of zoonotic disease to the human population. This study investigated the presence of neutralising antibodies against SARS-CoV-2 variants in serum samples (*n* = 142) from *E. helvum* bats captured between November 2017 and March 2019 in Makurdi, Nigeria. Faecal samples (*n* = 120) from the roost were collected in 2022 and screened for the presence of coronavirus RNA followed by genetic sequencing.

**Results:**

Virus neutralisation tests revealed 7.04% of the bat sera neutralised 2019-nCoV/Italy-INMI1, while 17.57% of the bat sera neutralised a SARS-CoV-2 Omicron BA.1 isolate. Partial genome obtained by next generation sequencing identified a lineage D *Betacoronavirus* from one faecal sample with 98.16% nucleotide sequence identity to sequences from *Eidolon helvum* collected in Cameroon in 2013. Epitope analysis of the spike protein sequence from the faecal sample showed conserved antigenic determinants shared with SARS-CoV-2.

**Conclusions:**

This study demonstrated that pre-pandemic sera collected from *Eidolon helvum* bats had neutralising activity against SARS-CoV-2 variants. Furthermore we detected the presence of lineage D *betacoronavirus* in this bat population that shared epitopes with SARS-CoV-2. This work contributes to our understanding of the complexities of coronavirus cross-reactivity. Characterizing bat coronaviruses is crucial to understand their zoonotic potential for spillover events due to bushmeat hunting practices.

**Supplementary Information:**

The online version contains supplementary material available at 10.1186/s12917-025-04938-y.

## Background

Coronaviruses belong to the *Coronaviridae* family comprising of diverse viruses known to infect humans and a broad range of animals. The *Orthocoronavirinae* subfamily is further divided into four genera: *Alphacoronavirus*, *Betacoronavirus*, *Gammacoronavirus* and *Deltacoronavirus* [1]. The *Betacoronavirus* genus has notably been responsible for zoonotic transmission of emerging infectious viruses resulting in disease in humans over the last two decades. Severe acute respiratory syndrome-coronavirus-1 (*Betacoronavirus pandemicum* /SARS-CoV-1; previously known as SARS-CoV) was detected in 2002 and infected over 8000 people, leading to more than 900 deaths worldwide [2]. SARS-CoV-1 is a member of the *Betacoronavirus* genus, subgenus *Sarbecovirus*, lineage B, and was likely transmitted to humans from masked palm civets sold in a market in Guangdong province in China [3]. In 2012, a related coronavirus initiated an epidemic on the Arabian Peninsula. The designated Middle East respiratory syndrome coronavirus (*Betacoronavirus cameli* /MERS-CoV) is also a member of the *Betacoronavirus* genus (lineage C) and causes severe respiratory disease in humans with high fatality. However, it is less efficient at transmitting among humans compared to SARS-CoV-1 [4]. Although most humans were directly infected with MERS-CoV by dromedary camels [5], human-to-human transmission was the cause of a substantial proportion of the cases [6]. MERS-CoV related virus has also been isolated from lesser bamboo bats (*Tylonycteris pachypus*) and Egyptian tomb bats (*Taphozous perforates*) [7, 8]. In 2017, SARS-related coronaviruses with high genetic similarity to SARS-CoV-1 were discovered in bat guano samples from horseshoe bat species (*Rhinolophidae*) indicating that bats are natural reservoirs of SARS-CoV-1 [9–11]. In December 2019, another *Betacoronavirus* in the *Sarbecovirus* subgenus, severe acute respiratory syndrome coronavirus 2 (SARS-CoV-2), was first reported in humans in Wuhan, China [12]. The World Health Organisation declared the outbreak (designated COVID-19) a global pandemic in March 2020 [13]. The origin of SARS-CoV-2 is still unknown, but the available evidence suggests that the likely natural reservoir are *Rhinolophus* species as highly similar coronaviruses have been identified in this bat species [14, 15]. SARS-CoV-2 has demonstrated an ability to cross the species barrier from humans to multiple animal species (reverse zoonosis). Since February 2020, a total of 42 animal species from domestic, captive, and wildlife species infected or exposed to SARS-CoV-2 have been reported, accounting for 929 animal cases in 40 countries worldwide [16].

Since the emergence of SARS-CoV-1, research on bat-borne coronaviruses has increased with heightened interest following the SARS-CoV-2 pandemic, contributing to our understanding on the global diversity of bat coronaviruses. Several studies have identified *Alphacoronaviruses* and *Betacoronaviruses* in African insectivorous and frugivorous bats including detections in Nigeria, Kenya and Guinea [17–21]. One species of bat studied, *Eidolon Helvum* (*E. helvum*), is widely distributed in Africa, hunted for human consumption, and known to harbour lineage D *betacoronaviruses* and viruses with zoonotic potential [21–24]. Furthermore, *E. helvum* bats are also of public health concern due to their adaptation to urban areas creating hotspots for human-to-bat interfaces, facilitating exposure of humans to pathogens in their excretions [22].

In this study we analysed serum and faecal samples from *E. helvum* bats in Makurdi, Nigeria, to contribute to our understanding of the impact bat coronaviruses may have on veterinary and public health.

## Methods

### Collection of serum and faecal samples

Wearing appropriate personal protection equipment, serum samples (*n* = 142) were collected from wild straw-coloured fruit bats (*E. helvum*) in Makurdi, Nigeria, between November 2017 – March 2018 and November 2018 – March 2019. These samples were part of a wider sample set collected to investigate the seroprevalence of Lagos Bat Lyssavirus antibodies in *E. helvum* [25]. The serum samples were heat-inactivated at 56 °C for 30 min and stored at 4 °C until required. Bat faecal samples (*n* = 120) were collected from the same roost between June and July 2022 and were stored in 1 ml DNA/RNA Shield (Zymo Research) and processed following manufacturers’ instructions.

### SARS-CoV-2 isolates

Two SARS-CoV-2 variants were tested in this study. SARS-CoV-2/human/Italy/LAZ-INMI1-isl/2020 (2019-nCoV/Italy-INMI1), GISAID accession number EPI_ISL_410545, was provided by the Italian Institute for Infectious Diseases (INMI) through the European Virus Archive GLOBAL project (EVA-GLOBAL, 008 V-03893) [26]. SARS-CoV-2/human/England/M02/2022 (Omicron BA.1), GISAID accession number EPI_ISL_18412401, was isolated from a nasopharyngeal swab from a UK patient. Both virus isolates were propagated in Vero E6 cells and used for serological testing. Laboratory work with SARS-CoV-2 was performed in licenced ACDP3 facilities at the Animal and Plant Health Agency.

### SARS-CoV-2 virus neutralisation test (VNT)

The virus neutralisation test (VNT) was performed on serum samples using a modified protocol from Loeffen et al., [27] and was performed in four replicates. The sera were diluted in cell maintenance media consisting of Dulbecco’s modified Eagle’s media (DMEM) (Gibco) supplemented with 2% foetal calf serum and 1% Penicillin/Streptomycin/Nystatin. A two-fold dilution series from 1/4 to 1/128 of each sample was prepared and diluted samples were mixed with 100 TCID_50_ of each SARS-CoV-2 isolate separately. Plates were sealed with gas-permeable membrane and incubated at 37 °C and 5% CO_2_ for 1 h, after which Vero E6 cells were added at a concentration of 5 × 10^5^/ml. Plates were sealed and incubated for five days and checked daily for the presence of cytopathic effects (CPE). A control plate with SARS-CoV-2 positive and negative sera was included for every run to verify the VNT. The positive control sera were produced at APHA by inoculating a pig with β-propiolactone inactivated 2019-nCoV/Italy-INMI1. The negative control sera were derived from healthy cats and dogs which were submitted to APHA pre-COVID-19 pandemic, for rabies serology testing (Pet Travel), and were tested previously to confirm the absence of neutralising antibodies against SARS-CoV-2. Presence or absence of CPE was recorded after five days and the neutralising antibody titre was calculated according to the Spearman-Karber method, expressed as 50% inhibitory concentration (IC_50_). The limit of detection of the test was 1.41 IC_50_ corresponding to all wells in the first dilution (1:4) producing CPE. A threshold for positive neutralisation was set at 2.83 IC_50_. This corresponded to an absence of CPE (i.e., virus neutralisation) required in all four wells of the first dilution to take into account any effects of non-specific neutralisation or experimental error.

### Pan-coronavirus RT-PCR and sequencing

RNA was extracted from the bat faecal samples using the MagMAX™core Nucleic Acid purification kit on the KingFisher™ Flex purification platform following the manufacturers’ instructions. The extracted RNA samples were tested by a two-step pan-coronavirus nested RT-PCR targeting a 440 bp fragment of the RNA-dependent RNA polymerase (RdRp) gene as described [28]. The first round used primers Hu-F 5’AARTTYTAYGGHGGYTGG 3’ and Hu-R 5’ GARCARAATTCATGHGGDCC 3’ and the QIAGEN OneStep RT-PCR Kit. The second round used primers Poon-F 5’-GGTTGGGACTATCCTAAGTGTGA-3’ and Chu 06-R1 5’-CCATCATCAGATAGAATCATCAT-3’ and Promega Go Taq^®^ G2 Flexi DNA Polymerase. Positive samples were visualised by gel electrophoresis and confirmed by Sanger sequencing. Sequence analysis was performed using DNASTAR^®^ Lasergene 15, the Basic Local Alignment Search Tool (BLAST) was used to identify the most closely related coronaviruses available on GenBank.

Whole genome sequencing was attempted for a single positive sample using next generation sequencing (NGS). Firstly, double-stranded cDNA was generated using sequence-independent, single-primer amplification (SISPA) [29, 30]. This involved first-strand cDNA synthesis using SuperScript IV (Invitrogen, Carlsbad, USA) followed by the NEBNext Ultra II non directional RNA second-strand synthesis module (New England Biolabs, Ipswich, MA, USA). Library preparation was performed using the Nextera DNA Library Prep Kit (Illumina, Cambridge, MA, USA) and sequenced using the MiSeq System (Illumina) according to the manufacturer’s instructions. Paired-end Illumina reads were assembled using a custom reference-guided alignment script (https://github.com/APHA-VGBR/WGS_Pipelines/blob/7f73c31629f483994b8aa366e157028abf69f824/RefGuidedAlignment_Public.sh) using the most closely related sequence identified by BLAST as the reference. Multiple sequence alignments were assembled using MAFFT v7.487 [31]. Phylogenetic analysis was inferred using the maximum likelihood method using IQ-TREE v2.1.4 [32] with a best-fit model applied using ModelFinder [33] with a phylogeny test of 1000 ultrafast bootstrap replicates [34], and visualised in MEGA 7.

### Spike protein epitopes

The Immune Epitope Database (IEDB) website (https://www.iedb.org/) [35] was used to search for SARS-CoV-2 spike protein epitopes. The search parameters were Organism: SARS-CoV2 (ID: 2697049, SARS2); Antigen: Spike glycoprotein [P0DTC2] (SARS-CoV2); Assay Outcome: Positive (T Cell, B Cell, MHC Ligand); Host: Any; Epitope Structure: Linear Sequence. A positive assay outcome signifies that experimental data in the database demonstrated that the selected linear sequences, when presented within its native protein context, has been experimentally validated to elicit an immune response through cell mediated antibody response. These assays inherently rely on the three-dimensional folding and implies that these sequences are part of, or contribute to, conformational epitopes. The database was accessed in May 2024. The top 50 epitopes with the highest number of experimentally positive assays were selected for investigation across the analysed sequences. The epitopes found in all analysed sequences were mapped against the spike protein sequences of the two SARS-CoV-2 strains used to evaluate seroconversion (Omicron BA.1 EPI_ISL_18412401 and 2019-nCoV/Italy-INMI1 EPI_ISL_410545), the Wuhan-Hu-1/2019 reference sequence (YP_009724390.1), bat *Betacoronavirus* in *E. helvum* from Cameroon (NC_048212.1, MG693169.1, MG693171.1, MG693172.1), and the partial spike gene sequence obtained in this study from *E. helvum*/Nigeria/37/2022 by performing a Multiple Sequence Alignment using MUSCLE in MEGA 11 [36]. The 50 epitopes screened in this study can be found in Supplementary Table S1.

## Results

### Neutralisation against 2019-nCoV/Italy-INMI1 and Omicron BA.1

Serological testing of 142 serum samples from *E. helvum* bats by SARS-CoV-2 VNT indicated the presence of antibodies with the ability to neutralise 2019-nCoV/Italy-INMI1 and Omicron BA.1 isolates. A total of 10 out of 142 (7.04%) bat sera had weakly detectable neutralising antibodies with titre ranges of 2.83- 8.00 IC_50_ against 2019-nCoV/Italy-INMI1. A subset of the samples with sufficient residual volume (*n* = 74) were also tested against Omicron BA.1, these included five out of the ten samples that had neutralising antibodies to 2019-nCoV/Italy-INMI1. results indicated that 13 out of 74 (17.57%) of the samples had antibodies with neutralisation activity to Omicron BA.1 with titre ranges of 2.83-32 IC_50_. Control plates were included with 11.31+/- 2 SD neutralisation titres obtained for the positive control sera against 2019-nCoV/Italy-INMI1 and no neutralisation detected with the negative control sera, verifying the assay. The positive control which was raised against 2019-nCoV did not have any cross neutralisation activity against Omicron where the titre was 1.41.

**Fig. 1:**
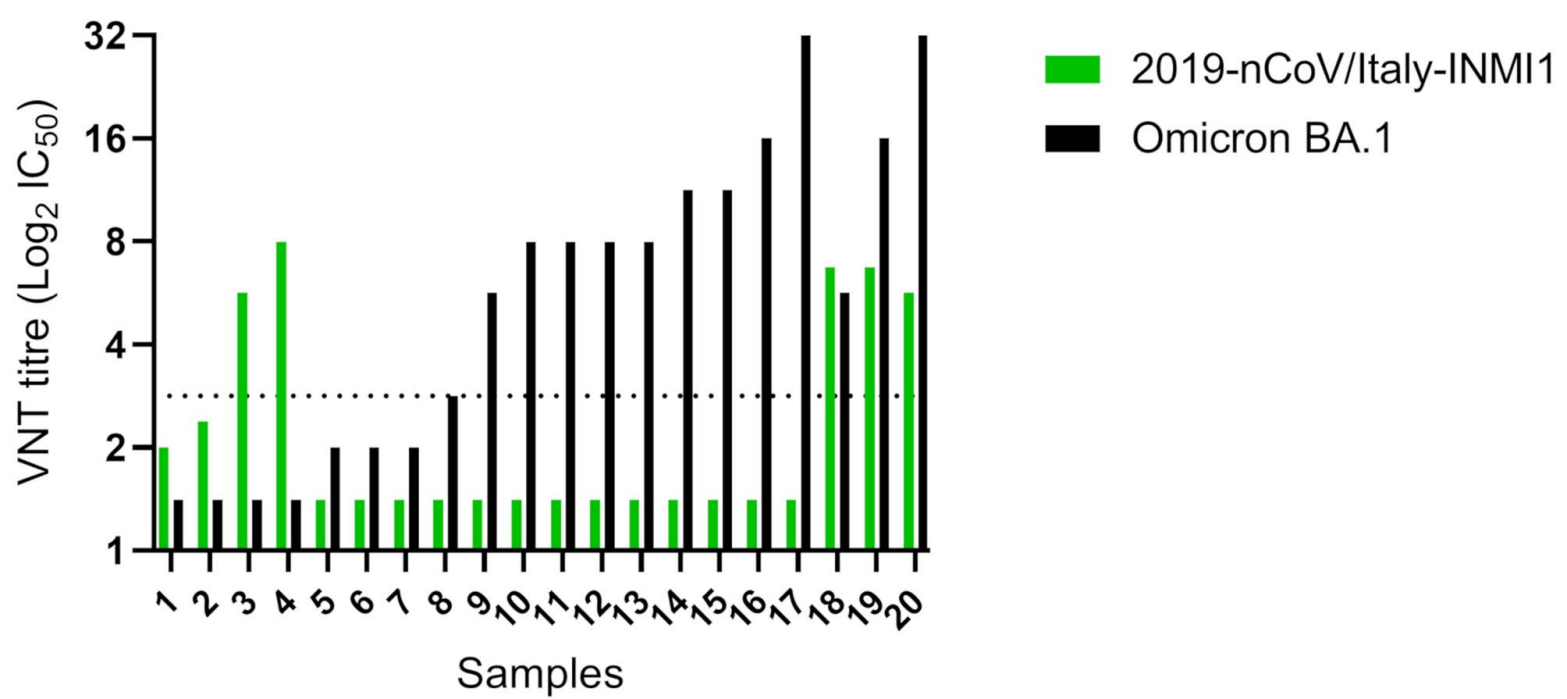
SARS-CoV-2 virus neutralisation titres of Nigerian bat sera. VNT titres of 20 serum samples with neutralising antibodies tested with both 2019-nCoV/Italy-IMNI1 (green bars) and Omicron BA.1 (black bars). The dotted horizontal line indicates the threshold for positive neutralisation (2.83 IC_50_)

In summary, out of the 74 serum samples tested against both isolates, 15 neutralised either one or both SARS-CoV-2 variants. Two samples neutralised only 2019-nCoV/Italy-INMI1, 13 neutralised only Omicron BA.1 and three neutralised both 2019-nCoV/Italy-INMI1 and Omicron BA.1 (Fig. 1).

### Detection of coronavirus RNA in *E. helvum*

To explain the neutralisation results obtained in this study, and assuming the colony would sustain the coronavirus infection in the population, bat faecal samples (*n* = 120) were collected from the same colony in 2022 and tested for the presence of coronavirus RNA. One sample was positive by PCR which generated a 394 bp sequence in the RdRp region of the ORF1ab gene; designated as *E. helvum*/Nigeria/37/2022. A BLAST search of the sequence obtained found it to be 99.75% identical to a lineage D *Betacoronavirus* collected in 2017 from *E. helvum* bats from Ghana (GenBank accession no. MT797298.1). In addition, this sequence had 99.48% and 99.24% identity to sequences from *E. helvum* from Rwanda in 2013 (GenBank accession no. KX285110.1) and Tanzania in 2018 (GenBank accession no. MT797339.1) respectively. Partial genome sequence (93.75% genome coverage) was obtained by NGS and submitted to GenBank (GenBank accession no. PP860750) with partial coverage in ORF1ab, Spike and M genes. BLAST search revealed that the partial genome obtained had 98.19% identity with bat *Betacoronavirus* from *E. helvum* collected in Cameroon in 2013 (GenBank accession no. NC_048212) and only 52.5% identity to SARS-CoV-2 (Wuhan-Hu-1/2019; GenBank accession no. NC_045512).

### Comparison of spike nucleotide sequences from *E. helvum*

**Fig. 2:**
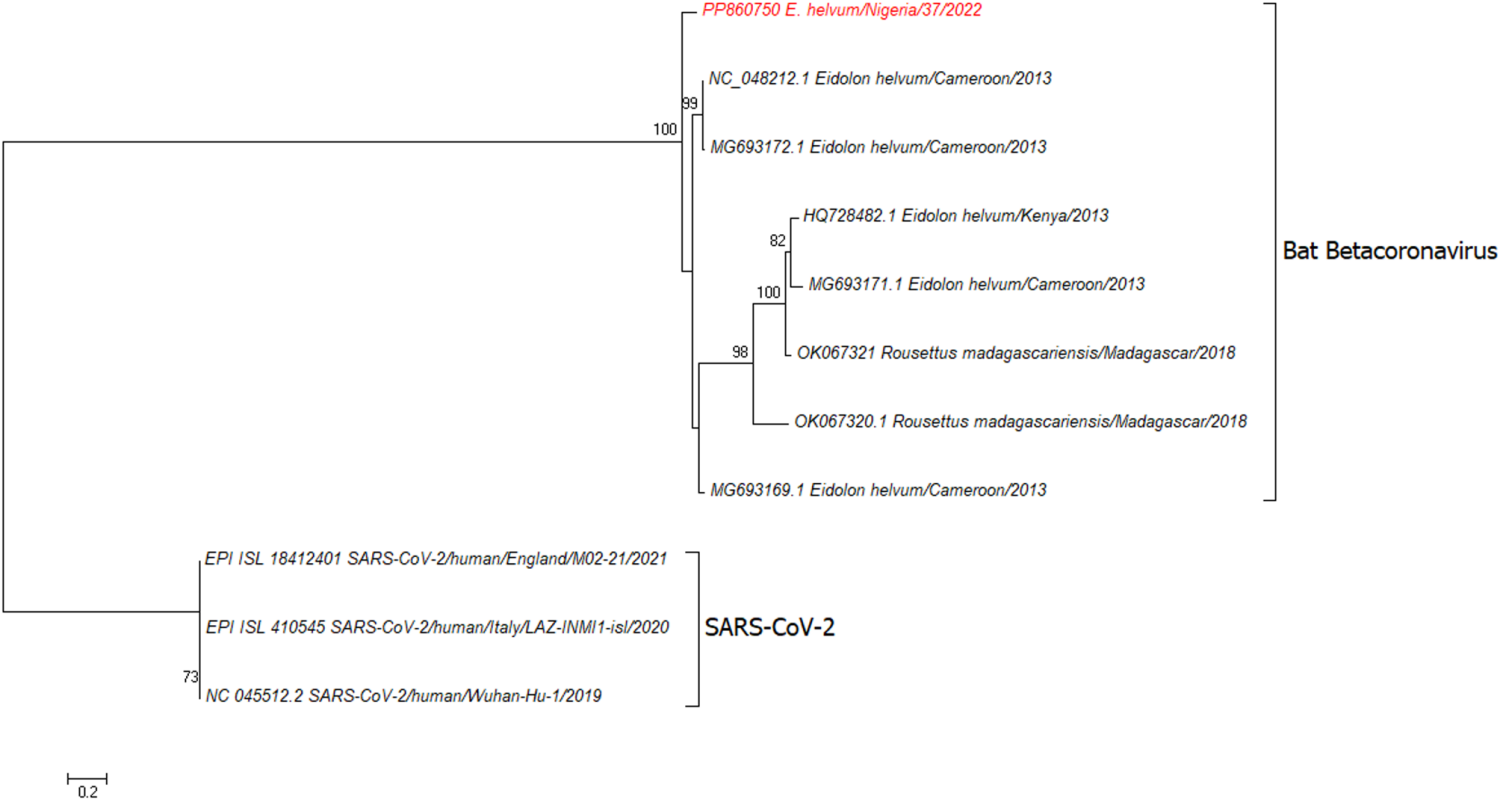
Maximum likelihood phylogenetic tree based on an alignment of 1736 nucleotides (including gaps) of the S2 region of the spike gene (positions 23700–25384 based on Wuhan-Hu-1/2019; GenBank accession no. NC045512). The maximum likelihood phylogenetic analysis was inferred using the TIM2 substitution model with empirical base frequencies (+ F) and four categories of rate variation (+ R4) with a phylogeny test of 1000 ultrafast bootstrap replicates. Bootstrap values greater than 70% are shown, figure generated using MEGA 7

Sequences with the highest similarity to the partial spike gene sequence from S2 region obtained from *E. helvum*/Nigeria/37/2022 by NGS in this study were identified by BLAST and added to the phylogenetic analysis (Fig. 2). These included available spike gene sequences of *Betacoronaviruses* from *E. helvum* and *Rousettus madagascarienis* from GenBank and were compared with SARS-CoV-2 Wuhan-Hu-1/2019, 2019-nCoV/Italy-INMI1, and Omicron BA.1 sequences. Nucleotide sequence comparisons identified bat *Betacoronavirus* in *E. helvum* from Cameroon in 2013 as the closest match at 88% identity (NC_048212.1) in the partial spike sequences compared.

### Assessment of spike protein epitopes

**Fig. 3:**
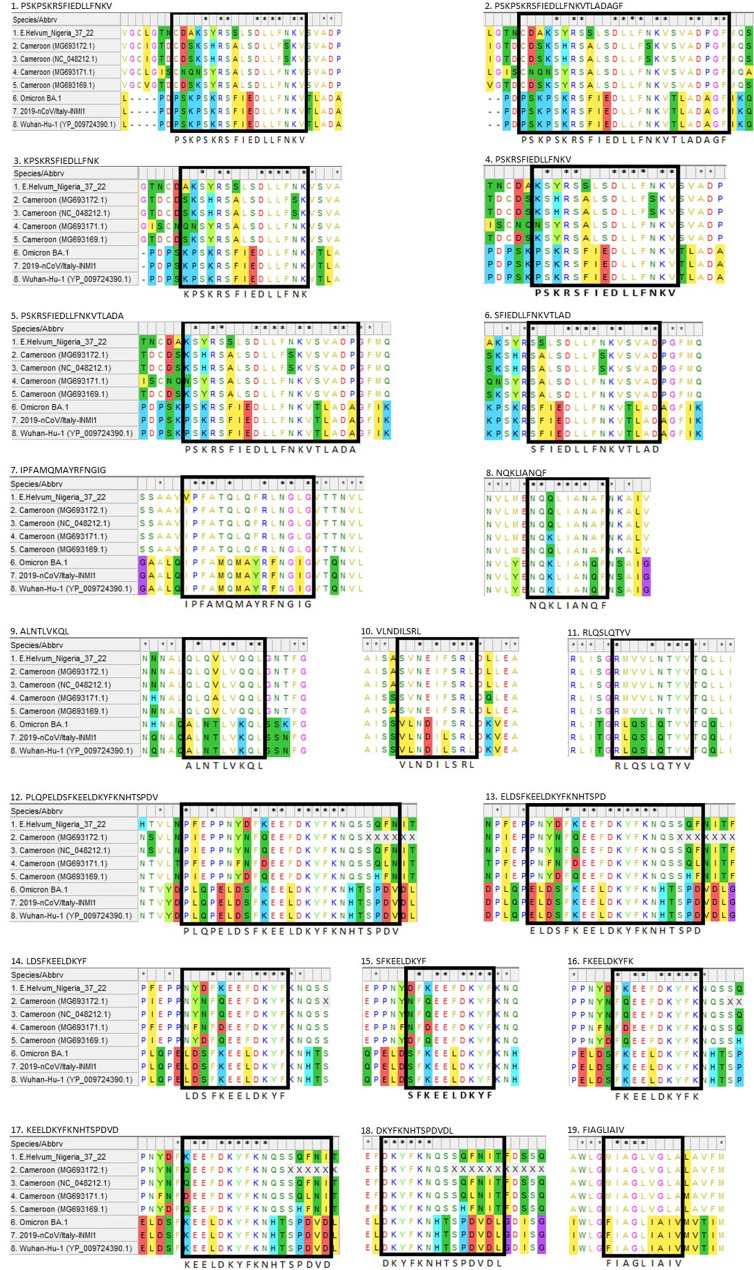
Mapped Epitopes. Multiple sequence alignment (MSA) of the mapped epitopes comparing sequences from Wuhan-Hu-1/2019 (YP_009724390.1), Omicron BA.1 (EPI_ISL_18412401), 2019-nCoV/Italy-INMI1 (EPI_ISL_410545), bat *Betacoronavirus* in *Eidolon helvum* from Cameroon (NC_048212.1, MG693169.1, MG693171.1, MG693172.1), and the partial spike gene sequence obtained from *Eidolon helvum*/Nigeria/37/2022 in this study. Residues are coloured according to their respective physico-chemical properties: Hydrophobic (A, F, I, L, M, V) in yellow; uncharged polar (N, Q, S, T, W, Y) in green; positive charge (K, R) in aqua blue; negative charge (D, E) in red; glycine (G) in fuchsia; histidine (H) in teal; cysteine (C) in olive green and tyrosine (Y) in lime. Residues exhibiting 100% conservation are denoted by an asterisk (*) above the alignment of each epitope and residues with a white background highlight the regions of the alignment with 60% or greater conservation among the analysed sequences at a particular location. The figure identifies the 19 epitope motifs that could have a role in antibody cross-neutralisation and are found across all sequences. The epitope localisation is denoted by a black square, and the epitope motifs are displayed below the alignment for reference

Nineteen experimentally confirmed epitopes from the IEDB database (Table S2) were identified in the spike protein sequences from the SARS-CoV-2 Wuhan-Hu-1/2019 reference strain and compared with 2019-nCoV/Italy-INMI1, Omicron BA.1, bat *Betacoronavirus* in *E. helvum* from Cameroon (NC_048212.1, MG693169.1, MG693171.1, MG693172.1), and the partial spike gene sequence obtained from *E. helvum*/Nigeria/37/2022. The mapping of the epitope motifs across the spike protein sequences revealed conserved regions and a significant abundance of amino acids sharing similar biochemical properties. All motifs presented in this study exhibit regions characterised by conservation levels of 60% or higher across the analysed sequences (Fig. 3). Epitopes were found in the fusion peptide 1, heptad repeats 1 and 2, and transmembrane domain. Notably, six epitopes exhibited a lysine (K) conserved exclusively between *E. helvum*/Nigeria/37/2022 and all SARS-CoV-2 isolates assessed. These epitopes and its respective residue and position number are: 12.PLQPELDSFKEELDKYFKNHTSPDV (K10), 13. ELDSFKEELDKYFKNHTSPD (K6), 14. LDSFKEELDKYF (K5), 15. SFKEELDKYF (K3), 16. FKEELDKYFK (K2), and 17. KEELDKYFKNHTSPDVD (K1). With the exception of an amino acid change at position 6 (L6F) in epitope 10. VLNDILSRL, all SARS-CoV-2 epitopes assessed were identical. The phenylalanine (F) at position 6 in epitope 10 was conserved among *E. helvum*/Nigeria/37/2022, bat *Betacoronavirus* from Cameroon (NC_048212.1, MG693169.1, MG693171.1, MG693172.1) and Omicron BA.1, while Leucine (L) was conserved between SARS-CoV-2 Wuhan-Hu-1/2019 and 2019-nCoV/Italy-INMI1.

## Discussion

Serum samples collected from *E. helvum* in Nigeria during 2017–2019 were able to neutralise two SARS-CoV-2 isolates; 2019-nCoV/Italy-INMI1 representing an early strain and Omicron BA.1 representing a variant of concern that emerged later in the COVID-19 pandemic. As the bat serum samples were collected before the pandemic, subsequent sampling of faecal specimens from the same roost were tested for the presence of coronaviruses. Detection of a lineage D *Betacoronavirus* in one *E. helvum* faecal sample suggested that these bats were infected with a lineage D coronavirus. Although the collection of faecal specimens occurred several years after the collection of sera it is assumed the colony would sustain the coronavirus infection in the population. This offers a possible explanation for cross-reactive antibodies against SARS-CoV-2 however serological studies specifically targeting lineage D *Betacoronavirus* would be required to confirm this.

Antibodies generated in response to bat coronavirus infection or exposure could neutralise when homologous residues (or residues with similar side chains) in the spike protein are present and have a suitable binding affinity [37]. The IEDB database was used to identify epitopes with conserved residues among SARS-CoV-2 strains and many of these conserved residues were found in the S2 subunit. Only four sequences of lineage D *Betacoronaviruses* from *E. helvum* were available in GenBank with sufficient genome coverage to compare their spike proteins. The alignment of the epitope sequences identified localised similarities among coronavirus spike proteins (Fig. 3) even though these bat *Betacoronavirus* sequences belong to lineage D, whereas SARS-CoV-2 is classified as lineage B. The cross-reactivity with SARS-CoV-2 isolates observed in this study could be due to the conserved residues identified in the epitopes found in the SARS-CoV-2 and Lineage D sequences (Fig. 3). Although the analysis was only based on the S2 subunit due to partial sequence obtained from *E. helvum*/Nigeria/37/2022, the S2 subunit has been shown to have cross neutralisation properties [38, 39], exhibit high conservation across different coronaviruses, and studies have shown immunogenic areas inducing cross-neutralisation [37, 40]. The shared epitopes with other pathogens and heterologous immunity are additional effects that may contribute to the neutralisation of unrelated pathogens [38]. The amino acid sequence of the N-terminal domain of the spike protein is highly conserved across the *Coronaviridae* family therefore it is plausible that exposure to bat coronaviruses could generate cross-reactive antibodies in SARS-CoV-2 serological assays [41]. Of the nineteen epitopes identified with conservation greater than 60%, six displayed a lysine residue present in both SARS-CoV-2 isolates and *E. helvum*/Nigeria/37/2022. Only one epitope (epitope 10) has a conserved phenylalanine residue, which is present exclusively in *E. helvum*/Nigeria/37/2022 and the Omicron variant. Although the function of these residues is unknown, this might explain the observed cross-neutralisation and the greater number of samples neutralising the Omicron variant. Considering that these in silico results are from linear peptide analysis, the conformation and the position of the contact residues in the biological structure may alter the affinity between these epitopes and neutralising antibodies [42, 43].

Bat serum samples collected from the same roost at the same time in Makurdi, Nigeria, were tested against Lagos bat lyssavirus (LBV) and a panel of pseudotyped viruses [25, 44]. The presence of neutralising antibodies against LBV (52%), Henipavirus (66.67%), Nipah virus (29.63%), bat influenza H17N10 virus (8.55%) and Ebola virus (0.36%) were reported. However, the authors did not observe neutralisation against SARS-CoV, SARS-CoV-2 (Wuhan) or bat coronavirus RaTG13 pseudotyped viruses. The sample selection for serological assessment with pseudotype viruses did not include the same samples assessed in this study and the contrasting results may be due to low level virus transmission and partial exposure to bats in the roost. An alternative explanation is that proteins beyond the spike protein can elicit neutralising antibodies. Cross-reactivities among coronaviruses have been observed in human serum samples collected pre-pandemic from sub-Saharan, Central and West African populations [45]. A large portion of the Sierra Leonean population had neutralising antibodies to SARS-CoV-1 and SARS-CoV-2 and a lower response to MERS [41]. A correlation between reactivity to nucleocapsid (N), Receptor Binding Domain (RBD) and the S2 subunit of the spike protein was demonstrated [41]. Similarly, human blood samples from Tanzania and Kenya, collected pre-pandemic also showed cross-neutralisation against SARS-CoV-2 [46]. Other studies have demonstrated the potential for cross-reactive antibodies to SARS-CoV-2 proteins following seasonal human coronavirus infection as well as SARS-CoV-1 and MERS [47–49].

## Conclusion

The *E. helvum* bats are hunted for human consumption and pathogens including viruses carried by these bats can potentially be passed onto bat handlers during processing. This study demonstrates the presence of antibodies in *E.helvum* sera that can neutralize SARS-CoV-2. Lineage D *Betacoronavirus* was identified in a faecal sample from this bat colony highlighting the potential for cross-reactive immunity among betacoronaviruses. The zoonotic risk and pathogenic potential of lineage D *Betacoronaviruses*, and the possibility of protection against SARS-CoV-2 following exposure, remains unknown. Further investigation is required to understand the impact bat coronaviruses may have on future outbreaks relevant for veterinary and public health.

## Electronic supplementary material

Below is the link to the electronic supplementary material.

**Supplementary Material 1**: **Table S1**: summary of top 50 SARS-CoV-2 Spike glycoprotein linear epitopes. Data were retrieved from the Immune Epitope Database (IEDB) using the database query parameters: Epitope Structure: Linear Sequence, Organism: SARS-CoV-2 (ID: 2697049, SARS2), Antigen: Spike glycoprotein [P0DTC2] (SARS-CoV-2), and Include Positive Assays. The query results were exported using the “IEDB Website Displayed” export type on May 15, 2024. The retrieved epitopes were then sorted by the number of assays, and the top 50 were selected for screening.

**Supplementary Material 2**: **Table S2**: List of epitopes identified in all compared sequences. The table lists the epitopes identified in all compared sequences. Each epitope is uniquely identified by an IEDB ID from The Immune Epitope Database (IEDB) website (https://www.iedb.org/) and classified according to a region of interest in the sequence. The functional region/domain within the spike is shown: spike subunit 2 (S2), fusion peptide 1 (FP 1), heptad repeat 1 (HR 1), heptad repeat 2 (HR 2), and transmembrane (TM). The number of independent assays confirming the epitope (# Assays) and the number of publications reporting the epitope with experimental data (# References) are also provided. The start and end positions of the epitope within the spike protein (UniProt P0DTC2 reference http://www.uniprot.org/uniprot/P0DTC2) are indicated. The epitope FKEELDKYFK was registered under two unique IDs, denoted by * for ID 2116377 and ** for ID 1397122.

## Data Availability

Partial genome sequence (93.75% genome coverage) was obtained by NGS and submitted to GenBank. The sequence data can be accessed under GenBank accession number PP860750.

## Abbreviations

SARS: Severe acute respiratory syndrome
CoV: Coronavirus
RNA: Ribonucleic acid
nCoV: Novel coronavirus
INMI: National institute for infectious diseases (Italy)
MERS: Middle east respiratory syndrome
COVID-19: Coronavirus disease 2019
GISAID: Global initiative on sharing all influenza data
EVA-global: European virus archive – global
VNT: Virus neutralisation test
DMEM: Dulbecco’s modified eagle medium
TCID_50_: 50% tissue culture infectious dose
CPE: Cytopathic effect
RdRp: RNA-dependent RNA polymerase
BLAST: Basic local alignment search tool
NGS: Next generation sequencing
SISPA: Sequence-independent, single-primer amplification
MAFFT: Multiple alignment using fast fourier transform
IEDB: Immune epitope database
MHC: Major histocompatibility complex
IC_50_: 50% inhibitory concentration
S2: Spike protein subunit 2
LBV: Lagos bat lyssavirus
RBD: Receptor binding domain
